# Navigating care together: developing a collaborative checklist to improve care for children with intellectual disabilities

**DOI:** 10.1186/s40900-025-00826-9

**Published:** 2025-12-10

**Authors:** Hayley Linda Trower, Chris Oliver, Leah E. Bull, Joanna J. Garstang, Kylie M. Gray, Heather Hanna, Kate Harvey, Ashley Liew, Sara Muller, Sheridan McDonald, Catherine Krupinski, Hayley Crawford

**Affiliations:** 1 Warwick Medical School, University of Warwick, Coventry, UK; 2 School of Psychology, University of Birmingham, Birmingham, UK; 3 Great Ormond Street Hospital, Great Ormond Street Hospital for Children NHS Foundation Trust, London, UK; 4 School of Nursing and Midwifery, University of Birmingham, Birmingham, UK; 5 Birmingham Community Healthcare NHS Foundation Trust, Birmingham, UK; 6 Southern Health & Social Care Trust, Craigavon, Ireland; 7 Evelina London Children’s Hospital, London, UK; 8 South London and Maudsley NHS Foundation Trust, London, UK; 9 Coventry, UK

**Keywords:** Intellectual disability, Coproduction, Clinical checklist, Parent/carer resource

## Abstract

**Background:**

While Patient and Public Involvement (PPI) has been increasingly integrated into research, its application in intellectual disability contexts can sometimes be overlooked due to perceived barriers. This study describes the collaborative development of The Behaviour Checklist, a tool designed to help parents/carers and clinicians identify and monitor causes of behaviours that challenge in children with moderate-profound intellectual disability.

**Methods:**

The study utilised consultation, collaboration, and user-controlled research, as outlined by INVOLVE. A Checklist Development Group of parents/carers, clinicians, and researchers reviewed and revised Version 1 of the checklist during monthly meetings over eight months. Feedback from two additional focus groups comprising parents/carers and clinicians was incorporated to refine usability, content, and relevance. The process followed the GRIPP2-SF reporting framework to ensure quality and consistency.

**Results:**

PPI contributed significantly to the checklist development by refining its purpose, functionality, item clarity, and visual format. For example, checklist items were revised to better align with real-world experiences, and guidance notes were simplified for lay comprehension. Feedback emphasised the checklist’s role as an aide memoire for identifying behavioural patterns and structuring clinical conversations. Parents/carers also highlighted its potential for monitoring changes, and supporting formal applications, while clinicians noted its utility in clinical decision-making. The finalised checklist was prepared for a pilot study, to assess its feasibility and acceptability.

**Conclusions:**

The involvement of parents/carers and clinicians positively influenced the checklist’s design, fostering a resource that balances scientific validity with practical relevance for service users and providers. PPI also strengthened relationships between stakeholders, paving the way for future collaboration. The checklist is ready for feasibility and acceptability testing and has the potential to improve outcomes for children with intellectual disabilities and their families by facilitating better understanding and management of behaviours that challenge.

**Supplementary Information:**

The online version contains supplementary material available at 10.1186/s40900-025-00826-9.

## Abstract (plain language summary)

Children with intellectual disabilities often display behaviours that challenge, such as self-injury or aggression. These behaviours can be distressing for the child and their family and can be difficult to manage. Understanding the reasons behind these behaviours are essential to improving care and reducing stress for everyone involved. This paper describes how we worked with parents, carers, and healthcare professionals to develop a checklist to help identify possible causes of these behaviours. This tool is designed for parents/carers to use at home and share with healthcare providers during appointments. The checklist may help parents/carers track their child’s behaviour and have meaningful conversations with clinicians. To ensure the checklist meets the needs of families and clinicians, we gathered feedback through group discussions and meetings. Parents/carers shared their lived experiences, while healthcare professionals contributed their clinical expertise. Together, we improved the checklist’s layout, language, and content to make it clear, practical, and relevant. By working together, we hope this tool will make a positive difference for children with intellectual disabilities and their families, improving communication and care in both home and healthcare settings.

**Supplementary Information:**

The online version contains supplementary material available at 10.1186/s40900-025-00826-9.

## Background

Patient and Public Involvement (PPI) is commonly referred to as “research carried out ‘with’ or ‘by’ members of the public rather than ‘to’, ‘for’ or ‘about’ them” [1]. PPI is integral to healthcare research in the UK and globally [2] and is often a funding requirement of research proposals [3]. PPI should improve the quality, integrity, relevance, and impact of research and ensure that patients’ needs and preferences are met [4–6]. Moreover, if patients and public are integrated into research, it is likely to involve greater interaction and more meaningful outcomes. There are differing ways in which patients and public might be involved in research, including advisory group membership, attending interviews or focus groups to help shape research, offering advice on research questions and methods, conducting interviews and data analysis, interpreting data, or contributing to research administration and dissemination [1, 7, 8].

The National Institute of Health Research in the UK (NIHR; previously INVOLVE; [1]) proposes three approaches to PPI when working with patients and public; namely consultation (requesting opinions and using them to influence decision making), collaboration (active and on-going throughout the research), and user-controlled research (patients and public invite professionals, and the design, implementation and dissemination is controlled by patients and public) [9].

People with intellectual disabilities are among those who are less able to advocate for themselves, making it essential that their needs are represented in research and healthcare decision-making [10]. Around 2% of the UK population have an intellectual disability [11, 12] which is characterised by impairments in IQ and adaptive functioning, including conceptual, social and practical life skills. People with intellectual disabilities experience poorer health outcomes and higher mortality rates than the general population [2], yet barriers such as limited professional knowledge, lack of confidence in supporting this population, and resource constraints often impact the quality of care they receive [13]. NHS England [2] has emphasised the importance of including those facing the greatest health disadvantages in PPI to ensure that research reflects their needs. However, for individuals with moderate to severe intellectual disability, direct participation in PPI can be challenging due to cognitive, communication and ethical considerations [14, 15]. Ethical concerns are particularly relevant when involving children in research, as issues such as capacity to consent, potential distress, and safeguarding must be carefully managed to ensure their well-being [16]. These considerations can make direct involvement impractical, reinforcing the need for alternative approaches.

Given these challenges, an alternative and highly valuable approach is to involve those who provide direct care and support – particularly parents and carers. As the primary service users of many healthcare tools and interventions, parents/carers play a crucial role in shaping practical, effective and meaningful resources. Parents/carers possess lived experience and insight into the day-to-day realities of supporting a child with intellectual disability, including recognising signs of distress, managing behaviours that challenge, and navigating healthcare and support systems. Their involvement in PPI ensures that research outputs, such as clinical checklists, are not only relevant and feasible but also tailored to the realities of caregiving. Although there are limited published accounts of PPI involving parents/carers of individuals with intellectual disabilities [7, 17–19], engaging parents/carers as key stakeholders offers an opportunity to bridge this gap [20, 21]. This study adopts such an approach, recognising parents/carers and clinicians as central to the development of a behavioural checklist designed to support children with moderate to severe intellectual disability. By integrating the perspectives of those who provide direct care, this PPI approach enhances the quality, usability and impact of the resulting tool.

The current paper describes the collaborative process that was involved in the development of The Behaviour Checklist during October 2021-October 2022, utilising the consultation and collaboration approaches and one aspect of the user-controlled research approach proposed by INVOLVE. The Behaviour Checklist is a clinical checklist that aims to help parents, carers and clinicians better understand behaviours that challenge in children with moderate to severe intellectual disability. The checklist aims to help parents, carers and clinicians identify the causes of behaviours that challenge so that the often harmful impact can be minimised for the child and others supporting them. The PPI described in this paper is an opportunity for service providers and service users to work together to develop a resource that will improve care and outcomes for children with intellectual disabilities.

Version 1 of the checklist was launched in January 2021 [22], and the development was based on more than 200 published peer-reviewed articles and decades of clinical experience, but did not include PPI. Version 1 has received positive anecdotal feedback from professionals and parents/carers and has been downloaded 1,415 times as of December 2024. A key reason for reviewing and revising the checklist was to include PPI, specifically welcoming collaboration from parents, carers and clinicians so that the checklist has a greater chance of being integrated into existing family life and clinical services.

## Method

With reference to the aforementioned approaches put forward by INVOLVE, the current study utilised ‘consultation’ and ‘collaboration’, and one aspect of ‘user control’ (design). The current paper utilises the short form GRIPP2-SF reporting checklist [23], the purpose of which offers evidence-based internationally agreed guidance on how to report PPI in research, to ensure quality, consistency, and transparency. The GRIPP2-SF reporting checklist consists of stages that the user should follow, and a brief description of each stage. The stages are aim, methods, study results, discussion and conclusions, and reflections/critical perspective. The inclusion of the GRIPP2-SF checklist guarantees that information about the context, process, and impact of public involvement is described, and is consistent with other published articles that have followed the same reporting procedure.

A Checklist Development Group (CDG) was established by the research team in order to collaboratively develop the checklist with stakeholders. The group was formed through direct contact made by the Principal Investigator or Research Fellow with academics and clinicians with expertise in intellectual disability, and parent/carers of children or adults with intellectual disability. Those contacted were already known to the research team and some were members of the research team. The CDG consisted of one parent/carer of a child with Angelman syndrome, severe intellectual disability and behaviours that challenge, who had previously contacted the research team offering their input, and one parent/carer of twin adults with fragile X syndrome, moderate-profound intellectual disability and behaviours that challenge who had previously taken part in research conducted by the research team. In addition, four academics, two consultant child & adolescent psychiatrists, one consultant paediatric dentist, one paediatric clinical psychologist, and two consultant paediatricians; all with experience of working with people with moderate-profound intellectual disability and behaviours that challenge were invited to be part of the CDG. Parents/carers were recompensed for their involvement.

The group met once a month for eight months via Microsoft Teams and, using Version 1 [22] as a template, discussed and refined the aims and objectives, target user group(s), checklist items, content, guidance for parents/carers and clinicians, additional documentation, formatting, grammar, comprehension from a lay perspective, and how it might fit into existing NHS services and home life with examples of differing scenarios involving behaviours that challenge. A first draft of the checklist was then developed by the research team in collaboration with the group through direct contact with stakeholders known to the research team and advertising via genetic syndrome support groups. The genetic syndrome support groups were those that provide services for people with rare genetic syndromes associated with intellectual disability. Given the complex needs of this population, advertising via genetic syndrome support groups ensured recruitment of parent/carers for whom the checklist was intended for. The aim of these new groups was to receive additional feedback on the revisions that were made after the first round of group discussions described above. A group of six parents/carers of children with moderate-profound intellectual disability met face to face on two occasions at the University of Warwick, lasting two hours on each occasion. A benefit of this new parent/carer group was that we would hope to receive feedback without the possibility of power dynamics that may have been present in the Checklist Development Group [1]. Parents/carers were recompensed for their involvement. The discussion focused on the checklist aims and objectives, usability, ease of understanding, usefulness, formatting, layout, and checklist items.

A group of nine clinicians (two GPs, two community paediatricians, three child and adolescent psychiatrists, a clinical psychologist, and a divisional medical director) met for 90 min on Microsoft Teams and discussed the same topics in the context of NHS clinical settings. Thereafter, the checklist was revised by the research team based on the additional feedback and a final draft was completed and sent to the original Checklist Development Group for final comments. Once final comments were received, reviewed, and implemented by the research team, a graphic designer produced a more visually appealing document, and a final version was emailed to all collaborators for final suggestions for minor edits.

## Results

### Overall outcomes

PPI during two phases contributed to the development of the checklist in several ways. This included discussing and identifying the checklist aims, discussing and identifying the intended checklist users, reviewing and identifying checklist items, contributing to checklist item guidance and notes, checking and amending the layout and formatting, checking and amending the content comprehension from a lay perspective, and contributing to publications.

A primary positive outcome as a direct result of PPI was the successful development of a checklist that has been co-produced by the service users and the service providers that it is intended to support. The Behaviour Checklist is now freely available to download from: https://cerebra.org.uk/get-advice-support/emotional-mental-health/behaviour-checklist/. A further primary positive outcome is that the checklist has been developed ready for pilot feasibility, acceptability, validity, and reliability testing.

### Instances of PPI impact

During phase 1, the discussions and revisions indicated by the two parents/carers focused on five areas: (1) Purpose; (2) Functionality; (3) Checklist Items; (4) Understanding and Clarity; and (5) Visual. Discussions with the parents/carers ensured that the checklist items (causes of behaviours that challenge) reflected real world experiences. For Purpose, the parent /carers agreed that the checklist would help to identify causes of behaviour and serve as an aide memoire that helps to monitor changes over time and to support conversations with clinicians. With reference to the checklist items, some of the original items were refined and/or reworded, some were separated into individual items, and ‘emotional outburst’ was removed (see Table 1). The parent/carers agreed that emotional outburst was experienced as a behaviour that challenges rather than a cause of the behaviour, that sensory problems should be more specific, and that social seeking and social avoidance should be separated because they present differently and may lead to different outcomes. Understanding and clarity was important to both parents/carers. They both suggested ways to alter the language for greater readability and update the guidance and notes that were associated with each checklist item. The parents/carers also agreed that the 20-page length of Version 1 might seem overwhelming to some parent/carers who are less comfortable with reading or who are under a lot of stress and experiencing heavy demands.

**Table 1:** Checklist items for both versions of the checklist

The checklist items (causes of behaviours that challenge)	
The Be-Well Checklist (version 1; 22)	The Behaviour Checklist (version 2)
Pain and discomfort	Pain and discomfort
Sensory problems/sensitivity	Sensory avoidance or sensory seeking
Anxiety	Anxiety
Low mood	Low mood
Sleep	Sleep difficulties
Emotional outburst	Impulsivity
Impulsive	Insists on sameness
Insists on sameness	Strong social seeking
Social differences	Strong social avoidance
Learned behaviour	Learned behaviour
Communication	Communication

The researchers and clinicians in the group agreed with the suggestions outlined above, although the removal of emotional outburst was not immediately actioned, and the decision was discussed at length over several meetings. Ascertaining the purpose of the checklist also required several meetings and benefitted from the multiple perspectives of parents/carers, clinicians and researchers to help pin down the ideal scenario for checklist completion, who it would benefit, and how it was of benefit. It was ascertained that the primary purpose of the checklist is to help parents, carers, clinicians and others systematically identify potential causes of behaviours that challenge in children with moderate to profound intellectual disability, and by providing a structured framework for discussion, it facilitates collaborative problem-solving, encourages new strategies for intervention, and ultimately aims to improve the well-being of both the child and those supporting them.

#### Sample quotes from parents/carers

Purpose:

I’ve actually found that quite useful to use … as a basic, going, well, you know how often does it happen. When do I expect it to happen again, and then I can see this sort of pattern…like an aide memoire with parents, but also at the beginnings of the furtherance of assessment. CK.

Functionality:

…the thing that really struck me is that the thing I’ve downloaded is 20 pages! And that just seems… and I don’t have a problem reading it or anything like that, and I imagine some people might find that in itself difficult, but it just seems very very long. It kind of needs to be doesn’t it because you need to understand it properly, but at the same time, it might be overwhelming, if there’s a lot going on and when am I gonna read it. SM.

Checklist items:

So where it says sensory sensitivity, it seems to only mention avoiding certain stimuli whereas I wondered if it could include seeking as well. SM.

Understanding and Clarity:

…it’s not going to fix things for you, it’s not going to show you what to do, it’s about just understanding better because they’re not being naughty, and they’re not. There is a reason, and you need to find it. It might be really difficult to unpick but you need to find it. SM.

Visual:

I think if maybe somebody was saying, if you had a YouTube video or you could discuss it before you even try to do it with somebody else or if you had an example in front of you, and you could see what it might look like and then you could home in on maybe just one rather than looking at two or three behaviours. CK.

During phase 2, the discussions and suggestions for revision focused on the same five areas: (1) Purpose; (2) Functionality; (3) Checklist Items; (4) Understanding and Clarity; and (5) Visual. Discussions with the parents/carers helped to further refine the purpose of the checklist, and several primary and secondary aims were suggested and developed. For example, parents/carers viewed the checklist as being primarily for gaining a better understanding of their child’s behaviour (including triggers and patterns in addition to causes), but also for educational purposes, to monitor change over time, and to support the completion of Disability Living Allowance (DLA) applications and Educational Healthcare Plans (EHCPs). In terms of functionality, the group agreed that the checklist functioned well as an aide memoire, and they would use it with the primary purpose in mind. Parents agreed with the formatting changes that had been made as a result of conversations with the Checklist Development Group in phase 1. With regard to the checklist items, a suggestion was made to include hyperfocus as a checklist item, or to be included with another checklist item. For example, a child may seek to self-soothe or self-regulate by hyper focusing on a task such as watching the washing machine. This is a behaviour that challenges because it isolates the child and is disruptive to home life. This suggestion was reviewed with the Checklist Development Group and discussed further with the parent/carer but was not included as a checklist item. This was because hyperfocus is often a core characteristic of autism and/or ADHD and thus not commonly regarded as a modifiable factor. As regards Understanding and Clarity, many points mentioned by the parents/carers during phase 1 were echoed, including a reiteration of, and agreement with the purpose. Additionally, parents/carers mentioned that the item ‘learned behaviour’ wasn’t very useful because they couldn’t see how it would help. The checklist language and reading level was not an issue for the group. With regards to the visual aspect of the checklist, it was agreed that the new layout made the most sense for ease of use.

#### Sample quotes from parents/carers

Purpose:

I think it can help to make things clearer in your head of how big an issue it is and how to track it see if it is improving or not because you forget. And then when you go to appointments, things can go out of your head and you can walk away and think oh I forgot to mention that. DB.

Functionality:

This all sounds lovely in an ideal world where you get appointments like that, but by the time I’ve done all of that, I’ve got a new behaviour that started. We only see the paediatrician every 6 months. So it would be like here’s 6 months of forms. MM.

If you’ve got no new behaviours, then presenting this with 6-month increments may be useful for building a bigger picture regarding ways of interventions. But as parents in families, you’re dealing with different behaviours, maybe you could present them as a caseload to your paediatrician. SM.

Checklist items:

It’s good to separate out the social avoidance and social seeking because my son seeks out adult company but he doesn’t like children’s company. DB.

Understanding and Clarity:

So, behaviours of concern. My son loves to flood the bathroom. He locks himself in there and turns the tap on and puts his thumb against the tap and sprays it everywhere. So would I fill this every week for flooding the bathroom? What about throwing toys down the stairs. Would I fill out a form for each behaviour? There might be 30 behaviours? RB.

Visual:

Could you compress the Likert scale into three and use traffic light colour coding – red amber and green? ON.

## Discussion

This paper describes the process of utilising PPI to co-produce a checklist for parents/carers and clinicians to use to identify and monitor the causes of behaviours that challenge in children with moderate-profound intellectual disability. The people involved in the PPI process were parents/carers of children with intellectual disabilities, clinicians who provide health care for people with intellectual disabilities, and researchers with expertise in intellectual disabilities. The key finding is that PPI positively influenced the development of The Behaviour Checklist according to five areas: (1) Purpose; (2) Functionality; (3) Checklist Items; (4) Understanding and Clarity; and (5) Visual. The positive influence of PPI is likely to have been influenced by the relationship between parents/carers, clinicians, and researchers, prior experience of PPI, and the checklist’s perceived relevance.

The parents/carers involved in developing Version 2 of the checklist had previously been involved in research PPI. This was beneficial because it meant that parents/carers were already familiar with research activities such as attending online meetings using Microsoft Teams, awareness of ethics and confidentiality, and importance of deadlines, thereby increasing confidence during PPI activities. Although prior experience of PPI was not necessary, it still had the potential to positively impact the conversations held between parents/carers, clinicians and researchers, as well as parents/carers’ expectations about project involvement. Despite these benefits, prior experience of PPI can introduce bias through experienced PPI members becoming more familiar with research processes, terminology and expectations which could lead them to align more closely with researchers’ perspectives potentially reducing the diversity or ‘lay’ perspective they offered.

Sometimes, during checklist development meetings, parents and carers referred to clinicians and researchers as the experts, despite their own lived experience. This suggests a perceived difference between them and the professionals, reflecting an imbalance in power. The NIHR [1] co-production guidance highlights the importance of power sharing in research, ensuring that all contributors, including those with lived experience, are valued as equal partners. In this study, the established relationships between parents/carers, researchers, and clinicians may have helped to reduce this imbalance. Without these relationships, the perceived difference may have been more pronounced, potentially leading to less productive discussions.

Conversations led by parents/carers also highlighted important features of the checklist from the perspective of the service user which would not have been possible without their input. For example, if parents/carers did not share their personal stories, then the checklist items may not have been amended. In particular, parents/carers recognised that the item ‘emotional outburst’ was a behaviour that challenges, rather than a cause, which led to its removal from the checklist. This was overlooked by the clinicians and researchers.

A further positive outcome was that collaborating with parents/carers did not compromise the scientific validity of the checklist. This is important to acknowledge, as some researchers may be hesitant to incorporate patient and public input in cases where they believe outcomes should be primarily informed by published evidence. In this study, rather than weakening the scientific foundation, PPI enhanced the checklist’s feasibility and relevance while ensuring it remained grounded in established research and clinical practice.

The experience of researchers was also a positive influence. All researchers involved had academic expertise in intellectual disabilities and behaviours that challenge, many of whom have worked with children and adults with intellectual disabilities during experimental fieldwork and many of whom have presented research at family conferences and met with families and children. Some also had clinical experience of working with children with intellectual disabilities, and some researchers had experience of PPI with the parents/carers and clinicians that were involved in the current project. It is likely that the pre-existing relationships between clinicians, researchers, and parents/carers would have positively influenced the level of trust and connection between all parties during phases 1 and 2 leading to more authentic conversations and greater levels of critical reflection.

Having reviewed Version 1 of the checklist prior to commencing PPI, parents/carers understood that the outcome of the checklist development would benefit them as well as other parents/carers with similar experiences, because it provides easily digestible information about their child’s behaviour, it prompts new thought processes, and enables them to monitor change over time. Behaviours that challenge can be distressing for all involved, and therefore, parents/carers were emotionally invested in co-producing a tool that can help to alleviate distress for their children, themselves and other families.

Each of the clinicians who were involved in the checklist development had an interest in supporting families and children who experience issues relating to behaviours that challenge. Thus, each clinician was able to recommend the checklist to service users and other service providers, and some were in a position to utilise the checklist themselves. It is probable that clinicians’ professional interest in the checklist would have positively influenced the conversations, leading to meaningful outcomes.

### Reflections/critical perspective

There are several reflections that may be beneficial for other researchers including PPI in their work. These reflections largely relate to the people involved in PPI, the PPI approach (in accordance with guidance from INVOLVE), reasons why PPI wasn’t used in the follow-up feasibility and acceptability study, and quantifying PPI.

Children with moderate to severe intellectual disability were not included as part of the PPI panel due to the communication challenges associated with this group which would have made meaningful participation in the project’s tasks unfeasible. Instead, parents/carers, as the primary service users of the checklist, were involved in its development. Their lived experience of supporting children with intellectual disabilities provided essential insights into the challenges associated with behaviours that challenge and helped shape a tool that is both practical and effective for those who will use it in real-world settings. Although we view this as a valid reason to not include children with intellectual disabilities on the PPI panel, we acknowledge that it is important to include people with intellectual disabilities in PPI in future resource development, especially where they would be direct users of research outputs. This could be achieved by collaborating with people who have less severe communication deficits, because they may still have insights that would be useful for understanding those with severe and profound differences as well as resourcing for the use alternative methods of communication.

The contribution from two parents/carers during phase 1 was invaluable, but it is possible that a broader perspective from more parents/carers would have enhanced the discussions. Many parents/carers of children with intellectual disabilities have a learning difficulty themselves, and many are from ethnic minority backgrounds where English is not their first language. They are also reported to have lower levels of well-being [13]. These population characteristics add to the barriers that already exist when recruiting participants and/or collaborating with patients and the public [24]. There can be resistance towards participating in research [25, 26], and this problem may also exist for PPI. Thus, it is important to cultivate relationships that build trust and confidence so that patients and public feel more capable and willing to engage in PPI [27].

A positive outcome regarding different perspectives among the PPI panel was that the phase 2 feedback differed between parents/carers and clinicians. Parents/carers favoured the checklist and identified several primary and secondary benefits, whereas some clinicians were concerned that the checklist would not be understood by some parents/carers. This perspective difference is reassuring because it indicates that there were honest conversations among parents and carers during phases 1 and 2. It also reinforces the need for non-professionals to be involved in research-related activity, to counteract the risk that parents/carers’ opinions and experiences could be overshadowed by the opinions and experiences of professionals. The potential for this risk was highlighted when a parent/carer commented that they were not the expert, even though they had lived experience. Despite a possible risk of power dynamics, capturing multiple perspectives was a positive and healthy outcome of the current project, which strengthens the efficacy of the checklist.

The PPI approach utilised for the current project aligned with guidance provided by INVOLVE [9], which proposes three approaches: consultation, collaboration, and control. Consultation and collaboration were distinct features of the checklist development in that the opinions of service users and service providers were requested and used to influence decision making, and there was active collaboration throughout the project. However, control was not as distinct a feature. Control refers to the design, implementation, and dissemination being controlled by patients and public. Whilst parents/carers were heavily involved in the design and dissemination, they did not have full control over the research process as this was maintained by the research team. Whilst we recognise the value of the ‘control’ model as a truly public-led approach, we did not adopt it for several practical reasons including limited capacity and support, feasibility, and mutual agreement. Our engagement instead with the ‘consultation’ and ‘collaboration’ levels demonstrated our commitment to producing the checklist with service users and service providers, with their involvement directly influencing key methodological decisions. This approach ensured that the research remained highly relevant, inclusive and grounded in lived experience of parents/carers of children with intellectual disabilities, while also maintaining methodological rigour and project feasibility [28].

A feasibility and acceptability study followed on from the current checklist development project. The study involved collecting quantitative and qualitative data about parents/carers use of the checklist at home and in NHS clinical settings. Finally, whilst we have reported the impact of PPI, we are unable to quantify the outcomes of parent/carer and clinician involvement. This challenge has been reported elsewhere [20, 29, 30]. Impact can be broadly described as encompassing effects and outcomes and is recommended as a valid reporting method [31].

## Conclusion

The current paper describes the PPI process that accompanied the development of The Behaviour Checklist, a tool for parents/carers and clinicians to better understand behaviours that challenge in children with moderate-profound intellectual disability. Parents/carers, clinicians, and researchers worked together to develop the checklist over several meetings and focus groups. The overall outcome was the development of a checklist suitable for parents/carers to complete at home with no or minimal support, which they can then take to a routine appointment with their clinician and use as a basis to structure conversations about behaviours that challenge. PPI improved the quality and relevance of the checklist for service users and service providers. A second outcome was that the checklist was ready to be completed by participants of a feasibility and acceptability study so that quantitative and qualitative data about the checklist could be collected and analysed. PPI had a positive impact on a tool that aims to be scientifically validated and utilised in home and clinical settings. A final outcome is that PPI strengthened relationships between parents/carers, clinicians, and researchers; thereby increasing the potential for future collaboration on similar projects.

## Supplementary Information

Below is the link to the electronic supplementary material.

Supplementary Material 1

## Data Availability

Data sharing is not applicable to this article as no datasets were generated or analysed during the current study.

## Abbreviations

PPI: Patient and Public Involvement
DLA: Disability Living Allowance
EHCPs: Educational Healthcare Plans
NIHR: National Institute for Health and Care Research
