# Supplementary material for: Navigating care together: developing a collaborative checklist to improve care for children with intellectual disabilities
Source: Res Involv Engagem. 2025 Dec 10;11:152. doi: 10.1186/s40900-025-00826-9 (PMC12729090; doi:10.1186/s40900-025-00826-9)
Supplement: Supplementary file 1 — Supplementary Material 1 [file 40900_2025_826_MOESM1_ESM.docx]

# GRIPP2 Short Form Checklist

1. Aim

The aim of patient and public involvement (PPI) in this study was to ensure that the Behaviour Checklist was co-designed with the people who would ultimately use it – parents, carers, and clinicians. Involving these stakeholders was intended to improve the checklist’s relevance, accessibility, and usability, and to ensure that the tool addressed real-world needs and experiences.

2. Methods

PPI was embedded throughout the study using both consultation and collaboration approaches. A Checklist Development Group (CDG) was formed, including two parent/carers and a range of clinicians. The group met monthly for eight months to review and revise the first version of the checklist. Feedback was also gathered from two additional groups: one consisting of parents and carers, and the other of clinicians. These sessions involved structured discussions around the checklist’s content, layout, guidance notes, and usability. Parents/carers were reimbursed for their time, and their feedback directly informed iterative revisions of the checklist.

3. Study Results

PPI had a substantial and direct impact on the development of the Behaviour Checklist. Parents/carers helped refine the checklist items, ensuring they reflected real-world experiences and were written in accessible language. Specific outcomes included the removal of the ‘emotional outburst’ item (which was identified as a behaviour rather than a cause), clearer guidance notes, and improved formatting to reduce overwhelm. Clinicians also influenced the checklist’s practical integration into NHS settings. One challenge was recruiting a broader and more diverse group of parent carers, particularly those from underrepresented backgrounds or with additional support needs. Despite this, the collaboration resulted in a checklist that stakeholders felt was usable, meaningful, and supportive.

4. Discussion and Conclusions

PPI had a meaningful influence on both the content and delivery of the Behaviour Checklist. The involvement of parents/carers, and clinicians ensured the final tool was not only grounded in evidence but also practical, relevant, and empathetic to the lived experience of families. The checklist became more user-friendly as a result of their input, with changes to language, layout, and item selection that would not have been achieved by researchers alone. A limitation was the small number of parent/carers involved in the initial development phase, due to recruitment challenges and the demanding circumstances many families face. Nonetheless, their contributions were significant, and later feedback from additional groups helped mitigate this limitation.

5. Reflections/Critical Perspective

The co-design approach fostered mutual respect and strong relationships between parents/carers, clinicians, and researchers. Many contributors had previous experience with PPI, which enhanced the quality of dialogue and reduced barriers to engagement. Feedback was candid and constructive, and changes were made collaboratively and transparently. However, challenges included difficulty recruiting a more diverse group of parents, particularly those with limited time, literacy, or language support. Some contributors occasionally referred to professionals as the “experts,” despite their own valuable lived experience. This suggested a perceived difference in roles, which the team aimed to gently challenge by fostering an inclusive and collaborative environment. Future projects will aim to broaden representation and explore more accessible formats for PPI involvement, such as creative or visual methods.
